# Biting Fibroma of the Lower Lip: A Case Report and Literature Review on an Irritation Fibroma Occurring at the Traumatic Site of a Tooth Bite

**DOI:** 10.7759/cureus.32237

**Published:** 2022-12-05

**Authors:** Philip R Cohen

**Affiliations:** 1 Dermatology, University of California, Davis Medical Center, Sacramento, USA

**Keywords:** traumatic, tongue, palate, lip, irritation, gingiva, fibroma, cheek, buccal, biting

## Abstract

Biting fibroma, an irritation fibroma or traumatic fibroma associated with a history of a prior lesion-related tooth bite or biting injury at the site, is a commonly acquired benign reactive lesion of the oral cavity. It is usually an asymptomatic, small, mucosa-colored, smooth, pedunculated or sessile papule. A biting fibroma is most commonly located on the buccal mucosa, followed by, in decreasing frequency, on the tongue or lip or hard palate or gingiva. It typically presents as a solitary lesion; however, it can appear as multiple lesions. Excision is the treatment of choice for a biting fibroma; however, the resolution of the lesion-associated chronic inflammation is also necessary to prevent a recurrence. The clinical history, lesion morphology, and pathology findings of an illustrative patient with a biting fibroma were included in this case report. An 80-year-old woman was described who developed a biting fibroma at the site of a tooth bite on her lower lip. An excisional biopsy not only confirmed the suspected diagnosis of a biting fibroma but also successfully treated her condition by removing the lesion; there was no recurrence. In conclusion, the diagnosis of a biting fibroma should be considered when a patient presents with a new intraoral lesion, particularly if associated with an acute injury or chronic inflammation of the site. Since the clinical differential diagnosis of a biting fibroma includes various other benign conditions and less common malignant neoplasms, a biopsy that removes the lesion may not only establish the diagnosis but also potentially provide adequate treatment.

## Introduction

Reactive conditions can present as an oral mucosal lesion [1]. An irritation fibroma, also known as a traumatic fibroma, is a reactive lesion of the oral cavity that appears as a localized, non-neoplastic, inflammatory hyperplastic papule of fibrous connective tissue [2-4]. When the etiology of the papule-precipitating event is a tooth bite or biting injury, the lesion may be referred to as a biting fibroma; therefore, that nomenclature shall be used in this paper.

Biting fibroma is a common lesion of the oral mucosa [5,6]. Although the lesion does not have racial or gender predilection, it is commonly observed in women over 30 years of age [4,7,8]. An excisional biopsy is usually effective not only for establishing the diagnosis but also for treatment [3,9].

A patient with a biting fibroma is described: an 80-year-old woman with an acquired papule on the mucosa of her lower lip. The location of the mucosal papule corresponded to a prior tooth bite site; the removal of the lesion, performed during the excisional biopsy, healed completely without recurrence of the oral lesion. The features of biting fibroma are summarized.

## Case presentation

An 80-year-old woman presented for the evaluation of an asymptomatic acquired lesion on her lower lip of two-year duration. Prior to the development of the new lesion, she recalled the nonintentional biting of her lip at this location. The site of the tooth bite, which resulted in an open wound, subsequently healed.

**Figure 1:**
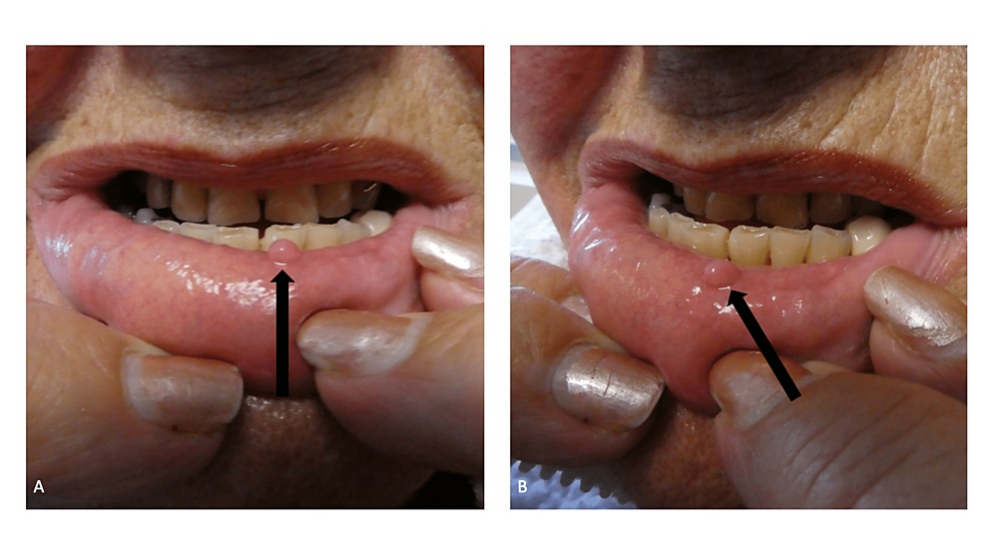
Biting fibroma presenting as a mucosa-colored papule on the mucosa of the lower lip The frontal view (A) and left-side view (B) of the lower lip mucosa of an 80-year-old woman show an acquired lesion of two years duration. The nontender, firm, flesh-colored, 3 × 3 millimeter smooth papule (black arrow) appeared after she bit her lip at the site

The patient assisted in the cutaneous examination of her lower lip. She gently everted the lip with the index and middle fingers from both of her hands. There was a painless, smooth, firm, flesh-colored, 3 × 3 millimeter papule on the mucosal portion of the lower lip, just left of the midline (Figure 1). An excisional biopsy was performed.

**Figure 2:**
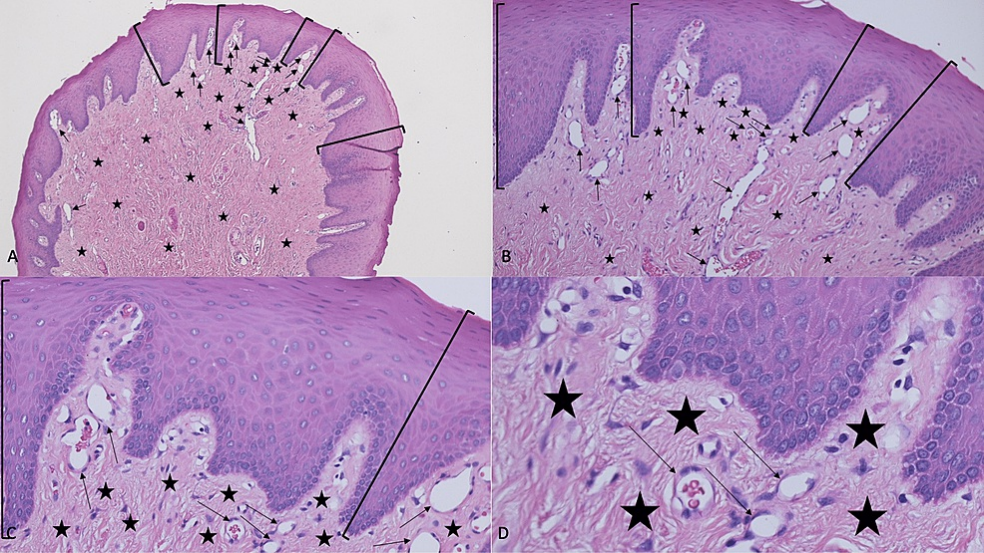
Pathologic presentation of a biting fibroma on the lower mucosal lip of an 80-year-old woman Distant (A) and closer (B, C, and D) views of the microscopic features of the biting fibroma from the patient’s lower lip are shown. There is slight thickening (acanthosis) of stratified squamous epithelium (black brackets). The underlying lamina propria contains collagen that is arranged in a radiating pattern (black asterixis). Numerous vessels (black arrows), some containing red blood cells, are also present in the lamina propria (hematoxylin and eosin stain: A, ×4; B, ×10; C, ×20; and D, ×40)

The microscopic evaluation of the tissue specimen showed collagenous fibrous tissue with vessels in the lamina propria. The arrangement of the collagen fibers had a radiating pattern, and some of the vessels contained erythrocytes. The overlying stratified squamous epithelium was slightly acanthotic (Figure 2).

The correlation of the clinical history, lesion morphology, and pathology findings established a diagnosis of biting fibroma. The biopsy site completely healed by second intention. During a follow-up period of 17 months, there was no recurrence of the lesion.

## Discussion

Biting fibroma is the most frequently acquired benign soft tissue lesion of the oral mucosa [6]. It can be observed not only in adults but also in children and elderly individuals [5,6,10]. In addition to self-biting, fibroma can result from other sources of localized trauma or chronic irritation [3,4,11-13].

Several terms have been used to describe a biting fibroma (Table 1) [1-20]. Indeed, the lesion is commonly referred to as either an irritation fibroma or a traumatic fibroma [1,4-20]. However, similar to the patient in this report, when the lesion is the sequelae of self-biting (whether intended or nonintentional), it is reasonable to include the mechanism of pathogenesis in the diagnosis: biting fibroma [8,13].

**Table 1:** Nomenclature used to describe biting fibroma

Terms	References
Biting fibroma	[8,13]
Fibroepithelial polyp	[8,13]
Fibrous hyperplasia	[8,14]
Fibrous nodule	[13]
Fibrous overgrowth	[15]
Fibrous polyp	[8,11]
Focal fibrous hyperplasia	[4,8,13]
Inflammatory fibrous hyperplasia	[13]
Irritation fibroma	[1-3,5-8,10,11,13,14,16-20]
Irritational fibroma	[8]
Localized fibrous hyperplasia	[8]
Localized fibrous overgrowth	[12]
Peripheral fibroma	[13]
Traumatic fibroma	[8,9,13,14]

The incidence of biting fibroma ranges from 1% (12 lesions per 1000 patients) to 15% (193 lesions per 1290 patients) [4,8]. Information from several studies on oral lesions published between 1986 and 2019 is summarized in Table 2 [1,2,5,6,9-11,16,19,20]. In one study, biting fibroma was the most observed oral lesion [20]. However, the rank number of biting fibroma compared to other oral lesions showed the diagnosis of biting fibroma to range from the second to the fifth (Table 2) [1,2,5,6,9-11,16,19,20]. In summary, compared to other conditions in patients evaluated for oral mucosal lesions, the occurrence of biting fibroma was observed to be 12.6% (853 of the 6792) of the lesions evaluated (Table 2) [1,2,5,6,9-11,16,19,20].

**Table 2:** Incidence of biting fibroma BF: biting fibroma; lis, lesions in study; M, men; NS, not stated; RN, rank number; W, women

Author	Year	Total number of lis	Total number of BF	Percentage of BF	RN of BF	Women with BF	Men with BF	Ratio of W/M	Reference
Bouquot and Gundlach	1986	1453	283	19.5	2	133	150	1.0:1.1	[19]
Zarei et al.	2007	172	44	25.6	2	24	20	1.2:1.0	[9]
Naderi et al.	2012	2068	288	13.9	4	120	168	1.0:1.4	[16]
Zuñiga et al.	2013	542	22	4.1	3	NS	NS	NS	[5]
Rivera et al.	2017	277	30	10.8	1	19	11	1.7:1.0	[6]
Hunasgi et al.	2017	460	45	9.8	3	31	14	2.2:1.0	[2]
Rivera et al.	2017	1000	102	10.2	1	77	25	3.1:1.0	[20]
Babu and Hallikeri	2017	659	30	4.6	5	12	18	1.0:1.5	[11]
Taweevisit et al.	2018	230	13	5.7	3	8	5	1.6:1.0	[10]
Blochowiak et al.	2019	208	26	12.5	3	NS	NS	NS	[1]
Total		7069	883	12.5		424	411	1.0:1.0	

There was a female predilection in two large retrospective studies that only included patients with biting fibromas. In one study of 124 patients (with 129 lesions), 77 were women, and 47 were men resulting in a women-to-men ratio of 1.6:1.0 [8]. The second study included 193 individuals: 136 women and 57 men, resulting in a women-to-men ratio of 2.4:1.0 [4]. However, a summation of the data from various biting fibroma studies shows that the ratio of women to men with this lesion is 1.0:1.0 (Table 2) [1,2,5,6,9-11,16,19,20].

Biting fibroma has been observed in children [4,5,8,10]. However, the onset age of a biting fibroma was older than 19 years for approximately 90% of the patients [4,8]. Indeed, most patients were in their fifth to sixth decade when they acquired a biting fibroma [4,8,11,16].

The duration of time that the biting fibroma was present prior to diagnosis was evaluated by one group of investigators; they had data from 112 of the 129 lesions in their study. The average duration was 19 months; the lesions had been present from one week to 13 years. However, the duration of the biting fibroma had been one year or less for nearly 75% (82 of 112) of the lesions [8]. The biting fibroma had been present for two years prior to the woman in this report seeking evaluation of the lesion.

Although the definition of a biting fibroma implies that it was preceded by prior trauma or irritation to the affected site, researchers are not always able to elicit a history of the causative event. In one study of biting fibroma, over 90% (175 of 193) of the patients related a history of local trauma [4]. Similarly, in another investigation in which this data was only available for 21 lesions, 95% (20) of the individuals had a history or either trauma (repeated, eight lesions, or non-repeated, six lesions) or an irritative event (such as removable dentures, three lesions; a fixed bridge, two lesions; or a natural tooth, one lesion); two of the patients who had lingual lesions also had a tongue habit that was the source of repeated trauma [8].

A biting fibroma usually occurs as an isolated event. A mucocele is an intraoral lesion that results from trauma to the minor salivary glands. Albeit rare, a nine-year-old girl with coexisting biting fibromas and a mucocele on her lower lip has been described [14].

Biting fibroma is typically asymptomatic. However, a study observed that 7.8% (10 of 129 lesions) were painful [4]. In addition to progressive enlargement, other biting fibroma-associated symptoms have included obstruction by the lesion during mastication and speech [13,17]. Occasionally, the lesion bleeds; this may be observed if the surface is ulcerated [9].

The greatest diameter of a biting fibroma is usually less than 1 centimeter [8,9]. Yet, lesions ranging from 1 to 2 centimeters are not infrequently observed [4,8,9]. Less commonly, fibroma is greater than 2 centimeters [4,9,13].

Similar to the patient in this report, the biting fibroma is usually a pedunculated or sessile growth with a smooth surface and mucosa-colored or yellowish-white papule. However, the surface can be hyperkeratotic or ulcerated [4,9,17,20]. Also, in patients with skin of color, the lesion can be brown to gray [13].

Biting fibroma may appear on any location within the oral cavity. The biting fibroma site observed in six studies is summarized in Table 3 [4,8,12,16,19,20]. Nearly 40% of biting fibroma were found on the buccal mucosa. The tongue (18%) and lip (16.5%) were the next most common locations. Thereafter, the hard palate and the gingiva (including the gum overlying the alveolar ridges of the mandible and maxilla), each 10%, were sites of lesion occurrence.

**Table 3:** Location of biting fibroma ^a^Biting fibromas observed on the mandibular ridge (19, 6.7%) and maxillary ridge (10, 3.5%) ^b^Biting fibromas observed on the gingiva (73, 25.3%) ^c^Biting fibromas observed on the alveolar ridge (nine, 4.7%) ^d^Biting fibromas observed on the gum (five, 4.9%) ^e^Biting fibromas observed on the soft palate (10, 3.5%) and oral floor (five, 1.9%) ^f^Biting fibromas observed on oral cavity sites that were not stated (16, 5.6%) ^g^Biting fibromas observed on the retromolar region (six, 3.1%) ^h^Biting fibromas observed on oral cavity sites that were not stated (34, 33.3%) BF, biting fibroma; H, hard

Authors	Barker and Lucas	Bouquot and Gundlach	Toida et al.	Naderi et al.	de Santana Santos et al.	Rivera et al.	Total
Year	1967	1986	2001	2012	2014	2017	
Total number of BF	171	283	129	288	193	102	1166
Buccal BF, number	62	78	42	113	119	40	454
Buccal BF, percentage	36.3	27.6	32.5	39.2	61.7	39.2	38.9
Tongue BF, number	25	57	66	35	25	7	215
Tongue BF, percentage	14.6	20.1	51.2	12.2	12.9	6.9	18.4
Lip BF, number	39	66	14	36	26	11	192
Lip BF, percentage	22.8	23.3	10.9	12.5	13.5	10.8	16.5
H palate BF, number	45	38	7	15	8	5	118
H palate BF, percentage	26.3	13.4	5.4	5.2	4.1	4.9	10.1
Gingiva BF, number	0	29^a^	0	73^b^	9^c^	5^d^	116
Gingiva BF, percentage	0	10.2^a^	0	25.3^b^	4.7^c^	4.9^d^	10.0
Other BF, number	0	15^e^	0	16^f^	6^g^	34^h^	71
Other BF, percentage	0	5.4^e^	0	5.6^f^	3.1^g^	33.3^h^	6.1
Reference	[12]	[19]	[8]	[16]	[4]	[20]	

Most individuals only have a solitary biting fibroma. However, albeit less frequently, patients with multiple biting fibromas have been described. In a retrospective study of 129 biting fibroma in 124 patients, multiple lesions were observed in three of the individuals. Two lesions were noted on either the tongue (tip and lateral border) of a 55-year-old woman or the hard palate of an 84-year-old woman. The third patient, a 79-year-old man, had three lesions on his buccal mucosa [8].

Multiple biting fibromas were also noted to suddenly appear on the tongue of a middle-aged Chinese man one week after an excisional biopsy of a benign lesion on his dorsal tongue. The biopsy of one of the five new lesions showed a biting fibroma; the site healed without the development of a subsequent lesion. Four months after the second biopsy, the investigators noticed a tendency toward gradual spontaneous regression of the new lesions [3].

Two concurrent biting fibromas were also reported on the lower lip of a nine-year-old girl who had a witnessed habit of lip biting; in addition, she also had a mucocele. All the lesions were excised; microscopic evaluation confirmed the diagnoses. After the surgical sites had healed, treatment was provided to ensure that recurrent injury to the location of the lesions did not occur; specifically, a lip bumper was placed on the woman’s lower lip for the management of the lip-biting habit [14].

The clinical differential diagnosis of a biting fibroma is diverse. Other reactive conditions of the oral cavity can have a similar morphology; these include fibroma, inflammatory fibrous hyperplasia, inflammatory gingival hyperplasia, peripheral giant cell granuloma, peripheral ossifying fibroma, and pyogenic granuloma [2,4,8,11,12,17]. In addition, other reactive and benign intraoral lesions that have been submitted by investigators evaluating biting fibroma patients include giant cell fibroma, mucocele, neurofibroma, polyp, salivary gland benign tumors, soft tissue mesenchymal tumors, and tumor (either benign or not otherwise specified) [4,8,13,17]. Oral squamous cell carcinoma can also mimic a biting fibroma [7]. A biopsy may be required to establish the diagnosis; the pathologic features of the conditions in the clinical differential diagnosis of a biting fibroma readily allow for the exclusion of lesions that morphologically mimic a biting fibroma.

The microscopic examination of a biting fibroma shows a connective tissue lesion in the mucosa that consists of dense collagen and a proliferation of mature fibroblasts. Chronic inflammatory cells (such as lymphocytes) may also be present; in addition, giant stellate cells may rarely be observed. The surface of the lesion can be hyperkeratotic or ulcerated or both secondary to the chronic irritation [2-4,8,12,13,17].

Two patterns of collagen arrangement in biting fibroma have been described: circular and radiating. The collagen pattern is influenced by the location of the lesion and the degree of irritation that the lesion has received. The circular pattern has been more commonly observed; it was usually associated with a mobile cheek lesion that was not fixed to the underlying bone and therefore less traumatized. In contrast, the radiating pattern was less frequently noted; it tended to appear in a lesion affecting mucosa overlying the bone that was immobile and received more trauma [2-4,8,12,13,17]. The woman in this report had a biting fibroma located on her lower lip, which showed a radiating pattern of collagen arrangement.

A group of researchers performed a light fluorescent microscope study of deparaffinized and unstained sections of 40 biting fibroma from either the buccal mucosa (27 lesions) or the lip (13 lesions) to determine the blue autofluorescence of the lesion’s collagen. They made two important observations. The intensity of the fluorescence increased with the age of the patient. Also, there was a positive correlation between the fluorescence intensity and the lesion duration [18].

In a follow-up investigation, the research group performed a polarized microscope study of formalin-fixed and paraffin-embedded sections stained with picrosirius red of 43 biting fibromas to determine the characterization of the collagen fibers of these lesions. They observed that there was not only an increased number of yellowish-orange and orange fibers but also fewer blue-green and green fibers both in patients greater than age 30 years and in biting fibromas of longer duration. The changes that they noted in the older lesions correlated microscopically with tighter packing and better alignment of the microfibrils, similar to that observed in mature collagen [15].

Based on their polarized microscope findings, the researchers concluded that younger biting fibroma primarily contained unpacked, poorly organized collagen whereas older lesions were composed of packed, well-organized collagen. Their observations also prompted them to recommend management based on the age of the biting fibroma and thereby its corresponding collagen characteristics. In a patient with an older lesion, not only stopping the chronic trauma but also surgical excision of the biting fibroma was necessary; however, in an individual with a younger biting fibroma, it was potentially possible for the lesion to spontaneously regress with only conservative treatment consisting of eliminating the chronic trauma [15].

The pathogenesis of a biting fibroma involves trauma or chronic inflammation or both. The trauma can be acute and isolated, such as a tooth bite of the lip. Alternatively, the trauma can be recurrent, such as chronic biting of the buccal mucosa of the cheek. Other potential causes of mechanical injury include calculi, dental prosthetics, dentures, foreign bodies, overextended borders of an oral appliance, overhanging margins of a dental restoration, and sharp spicules of the bones [3,4,9,11-13].

One group of investigators postulated that female hormones might be a cofactor in the pathogenesis of biting fibroma. In their study, they noted that a biting fibroma predominantly occurred in women (70%). They also mentioned that other reactive lesions of the oral cavity, similar to a biting fibroma, most commonly appeared during the first five decades when hormonal changes were most prominent. Therefore, they hypothesized that, in the presence of chronic intraoral injury in a woman, female hormones stimulated fibroblasts resulting in increased collagen production and accumulation [4].

A biting fibroma is a benign lesion; the malignant transformation of the lesion has not been described [7,20]. The genomic profile of the oral lesion may account for this observation. Researchers evaluated the gene expression pattern of antimicrobial peptides, growth factors, inflammatory chemokines and cytokines, matrix metalloproteases, and tumor suppressors in specimens from 15 biting fibromas and 15 healthy gingiva [7].

The researchers demonstrated significant gene expression elevation of *psoriasin* (*S100A7*, 11.3-fold) and *alpha-defensin* (*DEFA 1/3*, 14-fold) in the biting fibroma compared to the healthy gingiva. They also showed reduced gene expression of *matrix metalloproteinase-3* (*MMP-3*, fourfold) and numerous inflammatory markers: *interleukin 1 beta* (*IL-1β*), *interleukin 6* (*IL-6*), *interleukin 8* (*IL-8*), *tumor necrosis factor-alpha* (*TNF-α*), and *cyclooxygenase-2* (*Cox-2*). Finally, they noted that profound downregulation of *deleted-in-oral-cancer-1* (*DOC-1*, which is characteristically observed in proliferating malignant oral cavity tumors) was missing. They concluded that the lack of malignant transformation of a biting fibroma may be attributable to the concurrent missing downregulation of the tumor suppressor gene *DOC-1* and the overexpression of *S100A7* [7].

The management of a biting fibroma has two key components: the excision of the lesion and the prevention of recurrence by eliminating the etiology of chronic inflammation. The excision is usually performed using a scalpel. After hemostasis has been achieved, the surgical wound is often allowed to heal by second intention [1,3,4,8]. Electrical surgery or laser surgery can also be used to accomplish the removal of the biting fibroma [3,4,9].

Biting fibroma may develop at the site of the patient’s denture. A 46-year-old woman developed a large, 3.5 × 2.5 × 1.0 centimeter, biting fibroma (located on the lingual side of the retromolar pad) that originated at the posterior border of her maxillary denture [13]. In a study of 30 biting fibroma, 16.6% (five) of the lesions were denture-related [11]. When a biting fibroma is associated with an ill-fitting denture, successful treatment typically requires that the denture be realigned or remade [11].

There is a possibility of spontaneous regression of a biting fibroma in a patient who has a younger lesion if the source of irritation is removed; supporting evidence for this occurrence is based on fluorescence and polarized microscopy evaluation of the collagen arrangement in a biting fibroma. The collagen in an older lesion is more mature and organized; therefore, an older biting fibroma is unlikely to resolve spontaneously. However, in contrast to the older lesion, the collagen in a younger biting fibroma is poorly organized and may resolve without additional treatment if the source of irritation is eliminated. Hence, conservative management consisting of complete removal of the biting papilloma-associated irritation might be considered as an initial intervention if the lesion is newly acquired [15,18].

The recurrence of a biting fibroma is rare. However, several factors can contribute to provoking the recurrence of a biting fibroma. The lesion can persist and continue to grow if it has not been completely excised. Also, if the biting fibroma-initiating trauma persists or a new source of injury occurs at the site, the lesion can recur [4,11,17].

In a large study of 129 biting fibroma, there were no recurrences [8]. A 1% recurrence rate (two lesions) was observed in the postoperative follow-up period (ranging from one week to four years) of a study including 193 biting papilloma patients [4]. In a third study of 30 biting fibromas, only one case (3.3%) recurred [11].

A 13-year-old boy presented with a recurrence of the same lesion (which was located on the papillary region adjacent to the upper incisors) that had been surgically excised less than one year earlier; the proposed causes for the recurrent biting fibroma were the incomplete excision of the initial lesion or the nonremoval of the irritant (which was trauma to the affected area of the hard palate from occlusion from the mandibular incisors) or both. The recurrent lesion was completely excised; after the surgical site healed, the orthodontic management of the patient’s deep bite was successfully initiated by having the patient wear a removable anterior bite plane. After three months, the overbite decreased from 7.5 to 5.5 millimeters, and the intraoral appliance was discontinued; during the next nine months of follow-up, there was no recurrence of the biting fibroma [17].

The excisional biopsy site of the woman in this report had excellent healing by second intention. There was no recurrence of her biting fibroma.

## Conclusions

An irritation fibroma or traumatic fibroma is a commonly acquired benign reactive lesion of the oral cavity. The lesion is referred to as a biting fibroma, incorporating the causative etiology of the lesion in its diagnosis, when there is an established history of a prior tooth bite or biting injury at the lesion site. Most biting fibromas (nearly 40%) are located on the buccal mucosa; other intraoral sites include the tongue (18.4%), lip (16.5%), hard palate (10.1%), and gingiva (10.0%). The lesion typically presents as an asymptomatic, small, mucosa-colored, smooth, pedunculated or sessile, solitary papule. The treatment of a biting fibroma usually requires not only the removal of the lesion but also the resolution of the associated chronic inflammation; when the lesion is appropriately managed, recurrence is rare. An illustrative case of an 80-year-old woman with a biting fibroma that occurred at a tooth bite site on her lower lip is described. Her suspected diagnosis was confirmed, and the lesion was successfully treated with an excisional biopsy; the site healed by second intention, and there was no recurrence. In summary, if the diagnosis of a biting fibroma is suspected, a biopsy that removes the lesion may not only establish the diagnosis but also adequately treat the lesion.
